# Epilepsy in Turnpenny-Fry Syndrome: A Case Report

**DOI:** 10.7759/cureus.83923

**Published:** 2025-05-11

**Authors:** Rahul Khanna, Anudeep Surendranath, Saurabh Singhal

**Affiliations:** 1 Neurology, Neurology and Sleep Clinic, West Burlington, USA; 2 Neurology, CHI St. Vincent - Hot Springs, Hot Springs, USA; 3 Neurology, Opelousas General Health System, Lafayette, USA

**Keywords:** epilepsy case report, epilepsy genetics, epileptic seizure, intellectual disability (id), turnpenny-fry syndrome

## Abstract

Turnpenny-Fry syndrome (TPFS) is caused by a heterozygous mutation in the *PCGF2* gene on chromosome 17q12. A total of 15 cases have been reported to date. Of these, only two cases of TPFS have included a confirmed history of seizures. We report a new case of TPFS with epilepsy, which suggests that further studies of this rare disease are needed to fully understand the extent of developmental abnormalities associated with such genetic syndromes. This paper summarizes the clinical features to be aware of and the diagnostic genetic testing that can lead to the appropriate diagnosis, thereby contributing to the existing literature on this rare condition.

## Introduction

Turnpenny-Fry syndrome (TPFS) was first described by Peter D. Turnpenny in 2018 [1]. TPFS results from a heterozygous mutation in the PCGF2 gene situated at chromosomal locus 17q12 [2]. PCGF2 produces the polycomb group RING finger 2 protein, a transcriptional repressor essential for normal cell division, differentiation, and embryonic development [1]. Clinically, TPFS involves developmental and cognitive delays, poor growth, and a recognizable facial phenotype: frontal bossing, sparse scalp hair, underdeveloped cheekbones, short palpebral fissures, a small mouth, and distinctive dysplastic ears resembling those of a ‘satyr’ [1]. Patients frequently exhibit feeding difficulties, constipation, and malformations across neurological, cardiac, vascular, and skeletal systems [1]. A total of 15 cases have been reported to date. We report a new case of epilepsy in a patient diagnosed with TPFS. To date, only two cases of TPFS have been reported with a confirmed history of seizures [1]. The reported cases of TPFS with seizures only included seizure as a clinical symptom, while we report this case from an epilepsy standpoint. In this case, the patient initially presented to the clinic for epilepsy and was subsequently diagnosed with TPFS.

## Case presentation

A 35-year-old female was initially seen in clinic in April 2023 for epilepsy. She had a history of intellectual disability and epilepsy and had moved from Venezuela to Iowa, USA. She had her first seizure at five years of age. She and her mother are Spanish-speaking. The patient has severe intellectual disability and only limited communication capability. Her records, which were available in Spanish, were translated and reviewed.

She had an electroencephalogram (EEG) done in 2018 in Venezuela, which showed focal bursts of paroxysmal theta activity in the left posterior temporal-occipital area. As this EEG was done in Venezuela, records of EEG waveforms were not available. An EEG done at our center in May 2023 was a normal awake EEG but was limited due to electrode and muscle artifacts.

**Figure 1:**
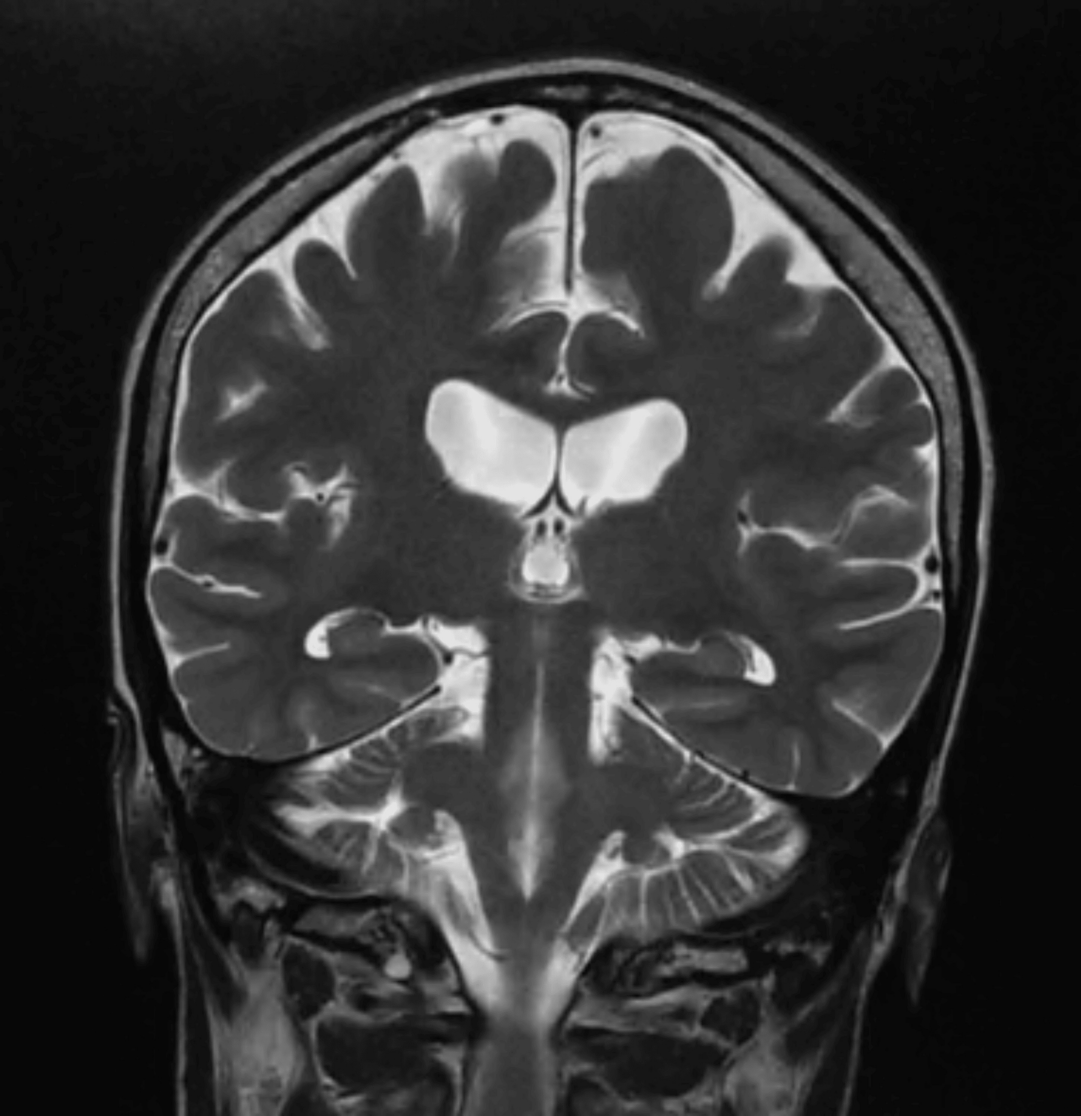
MRI brain shows normal hippocampus.

**Figure 2:**
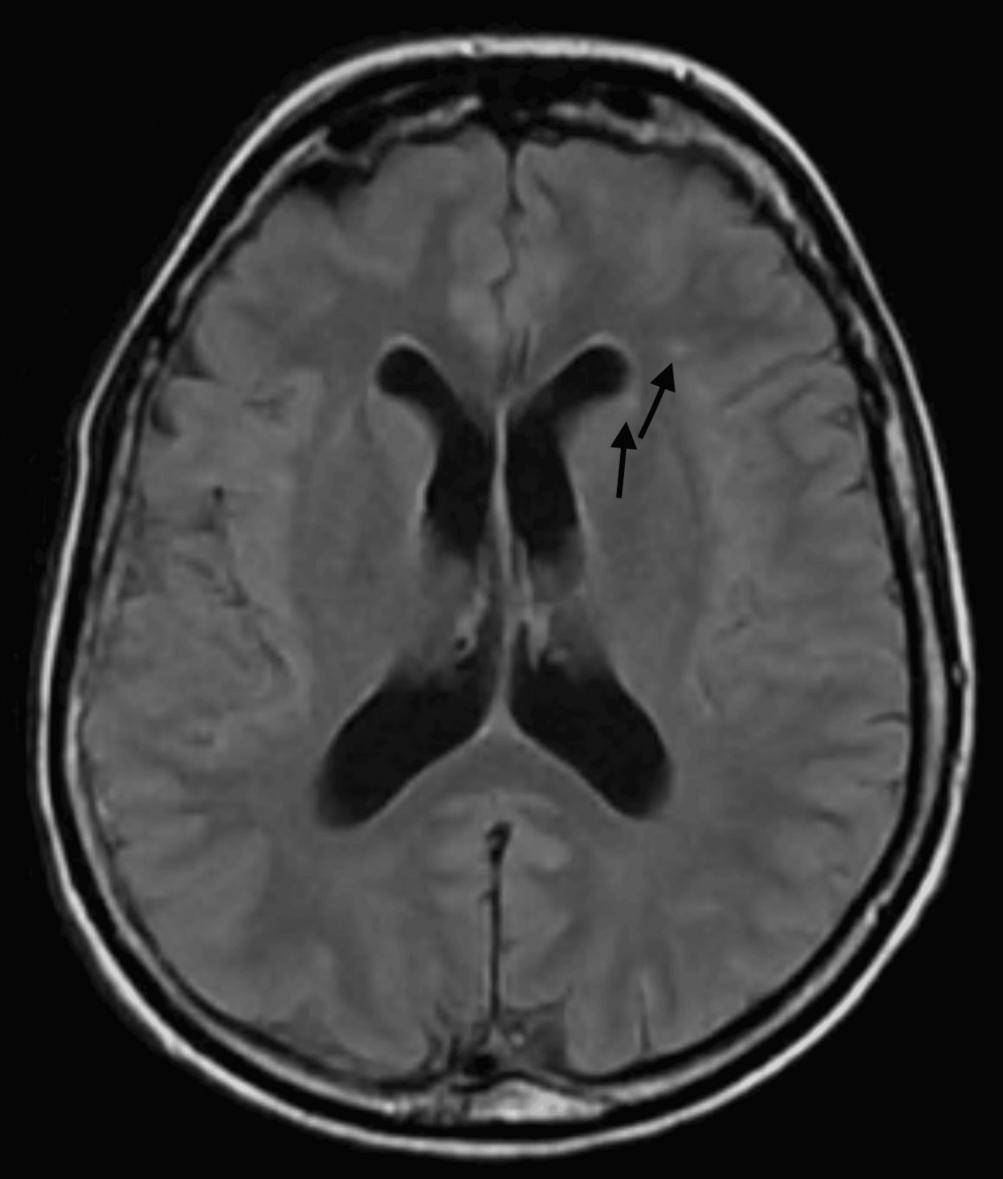
MRI brain showed incidental few punctate T2 and FLAIR subcortical white matter hyperintensities (marked by arrows). FLAIR: fluid-attenuated inversion recovery.

She had magnetic resonance imaging (MRI) of the brain done in the USA, which did not show any epileptogenic lesions. MRI brain findings (Figures 1, 2) showed a few incidental punctate T2 and FLAIR subcortical white matter hyperintensities. She has been on valproic acid 500 mg three times a day. Her last seizure was in March 2023. She has exhibited different seizure semiologies, including generalized tonic-clonic type events.

Because of her intellectual disability and epilepsy, she was referred to genetics. She was also followed up by psychiatry for her behavior. In August 2023, she had seizure-like activity for which she went to the Emergency Room (ER). Lamotrigine was started and slowly increased. She is currently on valproic acid 500 mg twice a day and lamotrigine 50 mg twice a day. She has been seizure-free on this regimen.

Based on her physical findings, she was referred to the genetics clinic at the University of Iowa, where whole-exome sequencing was performed. This revealed a pathogenic variant in PCGF2 associated with TPFS. The patient was also noted by the geneticist to have characteristic facial features that can be seen in TPFS.

## Discussion

In this case report, we identify a case of epilepsy with a genetic diagnosis of TPFS. A total of 15 cases have been reported to date, based on our literature review. We believe this to be the 16th case. To date, only two cases with confirmed seizures have been reported. With this case, epilepsy should be considered a possible manifestation in patients diagnosed with TPFS. Although the patient was noted to have focal EEG abnormalities on an EEG done in Venezuela, she responded well to broad-spectrum antiepileptic drugs such as valproic acid and lamotrigine.

Previous literature suggests that TPFS patients often have MRI abnormalities. MRI brain scans originally reported by Turnpenny et al. [1] showed mild enlargement of the lateral ventricles, polymicrogyria, and patchy to confluent white matter changes [1]. The severity of white matter changes varied, with the most pronounced alterations observed in individuals with polymicrogyria [1]. Our patient’s brain MRI showed only a few punctate T2 and FLAIR subcortical white matter hyperintensities, with no other abnormalities.

The genetic basis of TPFS is a heterozygous mutation in the PCGF2 gene on chromosome 17q12 [2]. In this condition, all reported mutations involve the Pro65 residue of PCGF2 [1], which lies immediately downstream of the N-terminal RING finger motif and is highly conserved across species and among other human PCGF family members [1].

PCGF2 plays a role in regulating the differentiation of cardiac mesoderm, which may underlie the cardiac anomalies observed in affected individuals [3]. Regular echocardiographic monitoring is recommended for all patients with PCGF2 mutations [1]. The brain and vascular abnormalities associated with the syndrome may result from disrupted PI3K-AKT signaling, a key pathway involved in growth, angiogenesis, and neural development [1]. Loss of PCGF2 function has been shown to downregulate PTEN, leading to enhanced AKT activation, increased HIF-1α levels, and upregulation of vascular endothelial growth factor (VEGF) [4].

As this report describes a single case, the findings may not be generalizable to the broader population of individuals with TPFS. Nevertheless, it adds to the existing literature on TPFS and epilepsy. Given the rarity of this condition, we hope this case report contributes to the growing clinical awareness and documentation of this entity, ultimately facilitating larger-scale studies in the future.

## Conclusions

TPFS, resulting from a heterozygous mutation in the PCGF2 gene on chromosome 17q12, is associated with a spectrum of developmental abnormalities; however, epilepsy has not been well documented as a feature of this condition. We report this new case of TPFS with epilepsy, which highlights the need for further research to better understand the full spectrum of developmental abnormalities associated with TPFS. Additionally, future multicentric prospective trials or studies can be planned to further corroborate these findings. When managing patients with epilepsy and developmental abnormalities, genetic disorders like TPFS should be considered, and genetic screening could be offered.
